# Development and Implementation of a Self-Care Plan for an Undergraduate Physiotherapy Curriculum in Switzerland: A Survey Study

**DOI:** 10.1177/23821205251374552

**Published:** 2025-09-15

**Authors:** Simone Zingg, Jorina Janssens, Irene Koenig, Patricia Wassmer, Angela Blasimann, Slavko Rogan

**Affiliations:** 1 School of Health Professions, Division of Physiotherapy, 69477 Bern University of Applied Sciences, Bern, Switzerland

**Keywords:** undergraduate physiotherapy students, self-care, undergraduate medical education, mental health, psychological resilience, mental disorder, behavior, coping, tool use behavior

## Abstract

**Background:**

Mental health, including well-being, coping strategies, and resilience, is a crucial aspect of overall wellness. In higher healthcare education, students’ mental well-being poses challenges for both learners and educators. While self-care is often emphasized in patient care during daily clinical practice, students’ own self-care needs are frequently overlooked. Incorporating self-care practices into healthcare education has been shown to enhance well-being and reduce burnout. This study aimed to demonstrate the impact of self-care plans on the mental health of undergraduate physiotherapy students.

**Methods:**

This quasi-experimental study involved two undergraduate physiotherapy cohorts (n = 192). The intervention consisted of developing and implementing a self-care plan. Both cohorts were instructed on the self-care plan; however, Cohort 1 received the plan after their first clinical placement, while Cohort 2 received it beforehand via an instructional video. A survey assessed self-care, perfectionism, self-doubt, and idealized images of everyday hospital life. Descriptive analyses were conducted for all outcomes, with a binomial test evaluating self-care perceptions and Pearson chi-square tests comparing cohorts and clinical placement timing.

**Results:**

Eighty students completed the survey. Students’ professional self-image in Cohort 1 became significantly less dependent on treatment success (χ^2^ = 10.9, *P* = .012), and coping with self-doubt improved after the second clinical placement (χ^2^ = 14.4, *P* = .001). After the second and third placement, 86% disagreed that clinical decision-making was difficult (χ^2^ = 93.4, *P* < .001). A significant association was found between Cohort 1 and 2, with substantially more students in Cohort 2 understanding what a self-care plan was (P = .002).

**Conclusion:**

Video instructions and information about a self-care plan impacted undergraduate physiotherapy students in some assessed parameters. To ensure effective implementation, enhancing cognitive learning and self-awareness through increased contact hours and more practice sessions seems essential.

**Trial Registration:**

Study registration number REES: ID: #19600.1v1.

## Background

Mental health is defined by the World Health Organization (WHO)^1^ as a state of well-being in which an individual realizes their own potential, cope with normal life stresses, work productively, and is able to contribute to the community. In summary, mental health is a holistic concept that includes well-being, coping, and resilience. However, according to the WHO^2^ 12% of the global population are suffering from a mental disorder. Approximately 14% of young people aged 10 to 19 suffer from mental health problems.^3^ Adolescents with mental health conditions are particularly vulnerable to social exclusion, discrimination, stigma (which affects readiness to seek help), educational difficulties, risk-taking behaviors, physical illness, and human rights violations.^3^

The fact that university students are more prone to mental health problems compared to young individuals in general has led to growing public concern in Western society.^4^ An estimated 35% of undergraduate and graduate students across various academic disciplines worldwide suffer from mental health problems or illnesses.^5,6^ In comparison, nursing students have an average prevalence of mental health disorders of 27.5%.^6^ Among undergraduate medical students, 50% are affected by depression and 67% by anxiety, with the comorbidities being more significant in later semesters than at the beginning of their studies.^7^ The mental health of higher education students poses significant challenges for both students and educators,^8^ particularly within health profession programs.

Therefore, the stressors experienced during physiotherapy education are a critical factor, significantly influencing students’ well-being, academic performance, and professional development, and thus warrant thorough examination. Physiotherapy students encounter several categories of stress throughout their training.^9^ Within academic settings, stress arises from the demanding curriculum, extensive workload, and limited time.^10^ Multiple studies identify the breadth of material, examination pressures, and the perceived difficulty of the program as significant contributors to anxiety. For instance, managing large volumes of information and navigating time constraints are major stressors for students in Australia and the United Kingdom.^10^ Similarly, psychological strain associate with academic assessments and coursework requirements.^11,12^ Workload and other factors of university life, including academic, financial, clinical, faculty, administrative, personal, research, and drug-related issues, were identified as stressors.^13^ Francis and Naftel^13^ identified academic stress (examinations, grades, workload) as the most significant issue. In clinical placements, stressors predominantly relate to patient care responsibilities and professional integration. Exposure to patient suffering and death has been identified as a critical trigger, alongside challenges in interactions with clinical staff.^9^ Additional research by Whiteside et al and Bennion et al underscores issues such as bullying, inadequate supervisory support, and the intricacies of patient care as significant sources of stress.^14,15^ Collectively, these findings underscore the dual impact of academic demands and clinical experiences on the stress landscape of physiotherapy students during their professional education.

These insights into the stressors faced by physiotherapy students naturally lead to an exploration of the strategies they employ to navigate and manage these challenges effectively. Physiotherapy students employ various positive strategies to manage the stress associated with academic and clinical training, as highlighted in several studies.^16–18^ In academic contexts, students often engage in active problem-solving strategies, such as planning, information gathering, and positive reframing, to navigate coursework and examinations effectively.^17,18^ Emotion-focused strategies are also prevalent, with acceptance, positive reappraisal, humor, and, in certain cases, religious solace frequently reported.^18,19^

During clinical placements, social support from family, peers, and clinical educators emerges as a key coping mechanism, complemented by stress-relief activities such as exercise and recreational breaks.^9,16^ Notably, an optimistic attitude appears particularly adaptive to academic challenges. However, a minority of studies document maladaptive coping strategies, including avoidance and substance use, which are associated with poorer outcomes.^17^ Therefore, self-care is an important approach for maintaining or enhancing well-being and for mitigating adverse effects.

Self-care agency refers to an individual's capacity to recognize their needs and to identify and undertake appropriate self-care actions.^20,21^ Research has indicated that effective self-care practice is intrinsically motivated and involves self-awareness, self-compassion, the practice of altruism and the implementation of a variety of strategies across physical, social, and inner self-care domains.^22^ Preparing students for their future work in health care requires to be able to take care of themselves and be aware of personal needs.^23^ Only healthy and balanced health care professionals can respond appropriately to the needs of patients and provide them with comprehensive care.^23^ However, Golz and colleagues^24^ identified deficiencies in current health care professions education institutions and emphasized the importance of students learning to manage themselves to reduce stress. It seems that the importance of self-care is often underestimated in current curricula, as students learn how to teach patients to self-manage, but the transfer to themselves is usually missing.^24^ In contrast, implementing self-care practices into the education of healthcare students has been shown to have a positive effect, enhancing their well-being, and reducing burnout.^25^ A 12-week online mindfulness and self-care program decreased stress levels and increased mindful attention awareness and self-compassion in healthcare students and faculty,^26^ while incorporating self-care activities into undergraduate psychiatric-mental health nursing courses similarly proved valuable in reducing burnout and improving student outcomes.^27^ Moreover, integrating a self-care assignment into core nursing coursework significantly increased students’ self-care practices, enhanced their knowledge of stress management, and supported overall well-being.^28^

So far, the undergraduate physiotherapy program at Bern University of Applied Sciences, School of Health Profession, Division of Physiotherapy (BFH-DPT) offers a mentoring program for all enrolled students, featuring individual discussions for a personal and performance-related assessment of the students to make them aware of their strengths and weaknesses. However, the promotion of self-care management was not integrated into the BFH-DPT curriculum until 2023, neither within taught modules nor through formal offerings. During the Coronavirus disease (COVID-19) pandemic, the number of students withdrawing from BFH-DPT programs increased from 1.13% to 2.17%. Considering the rising numbers of dropouts and the COVID-19 pandemic's negative impact on mental health of young adults, addressing the topic of mental health is warranted.

Therefore, a self-care plan was designed to promote self-efficacy regarding mental health. The self-care plan encourages individuals to incorporate supportive habits regarding physical, emotional, and social needs into their daily lives to recharge energy, promote self-awareness, alleviate emotional burdens, and enhance well-being and overall quality of life. Consequently, the purpose of this study was to investigate the impact of the self-care plan on the mental health of undergraduate BFH-DPT physiotherapy students during their clinical placement.

## Methods

### Physiotherapy and the Mentoring Program in the BSc Physiotherapy Curriculum

The Bachelor of Science in Physiotherapy program at BFH-DPT is currently structured as a 3-year, full-time course. Students complete their first clinical placement at the end of the third semester, lasting 10 weeks. These placements take place in hospitals or private practices. The second clinical placement occurs at the end of the fourth semester, also for 10 weeks. The third placement is scheduled at the beginning of the fifth semester, followed by the fourth placement at the start of the sixth semester, each lasting 10 weeks. Between the clinical placements, students participate in academic modules delivered both on and off campus, integrating theoretical knowledge with clinical practice. In the Bachelor of Science in Physiotherapy program, each student is individually supported by a mentor from the academic faculty. The mentoring program is systematically organized, with mentors and students scheduling one mandatory mentoring session per semester. These sessions are guided by structured conversation frameworks, which serve as preparation and a basis for discussion.

The primary goal of the mentoring program is to support students throughout their entire learning journey, fostering their development toward professional competency. Mentors are available as points of contact for the duration of the program and encourage students to engage in self-reflection, critical thinking, and responsibility. They assist students in identifying their strengths and weaknesses, setting learning objectives, exploring professional career opportunities, and addressing self-care.

### Study Design

This research project was designed as a quasi-experimental study with two non-randomized study cohorts (n = 192 in total). This current study was conducted at BFH-DPT in Bern, Switzerland. Survey on mental health and self-care were administered to two undergraduate physiotherapy cohorts (Cohort 1: n = 91¸ Cohort 2: n = 101). The study adhered to the reporting guidelines for observational studies^29,30^ and was registered at the Registry of Efficacy and Effectiveness (REES: ID: #19600.1v1). This study followed the SQUIRE-EDU guidelines (Standards for Quality Improvement Reporting Excellence in Education).^31^ By adhering to these guidelines, the study provides a clear, structured framework for understanding the impact of self-care education on physiotherapy students and the effectiveness of the interventions used.^32^ The reporting of this study conforms to the Revised Standards for Quality Improvement Reporting Excellence (SQUIRE 2.0) (please see Appendix).

**Figure 1.**
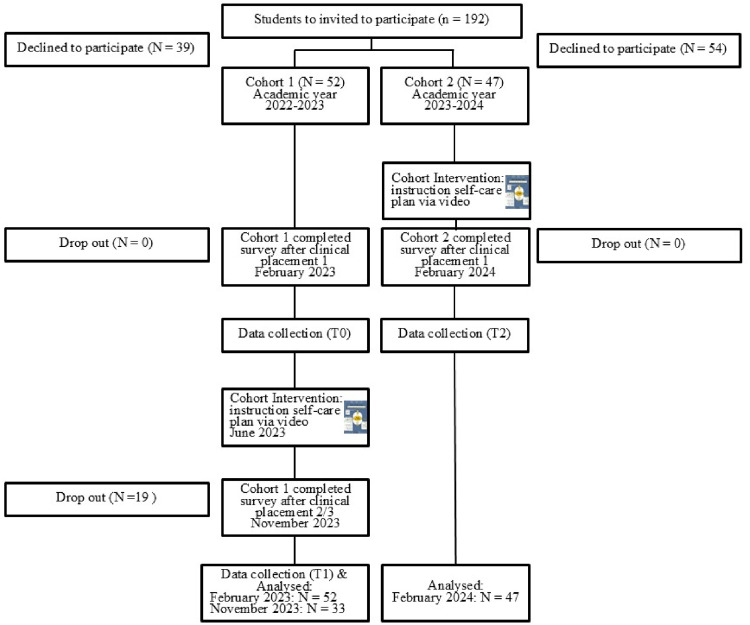
Presents the Survey Process.

Cohort 1 comprises students in the second year of their program who had completed their first clinical placement. These students participated in an initial survey (T0) conducted in February 2023. At this point (T0), the students did not receive any instruction or guidance related to the self-care plan. A follow-up survey (T1) was conducted in November 2023, during their third year, after completing their third clinical placement. By this point (T1), the students had already received information and guidance regarding the self-care plan. Cohort 2 consists of students in the year below Cohort 1. Their first survey (T2) was conducted in February 2024 after completing their first clinical placement. Unlike Cohort 1, these students received instructions on the self-care plan prior to beginning their first placement (see Figure 1).

The objective of the study was to administer a survey to Cohort 1 following their first clinical placement (prior to receiving any instruction on self-care planning) and again after their third placement (subsequent to receiving such instruction). The responses from these two time points within Cohort 1 were then analyzed and compared. In contrast, Cohort 2 completed the survey only after their first clinical placement, having received self-care plan instruction in advance. This design enabled a comparative analysis between the post-placement responses of Cohort 2—with prior exposure to self-care planning—and those of Cohort 1 following their initial placement without such preparation.

The survey using Wooclap (wooclap.com) took approximately 6 min to complete, with access available for 30 days.

### Intervention of the Self-Care Plan Within the Physiotherapy Bachelor's Program

The intervention involved the development and implementation of a self-care plan designed to support the mental health of undergraduate physiotherapy students. This process was initiated by a series of discussions held by a group of experts at BFH-DPT, who collaborated with an international expert renowned for her work in supporting student mental health.

**Figure 2.**
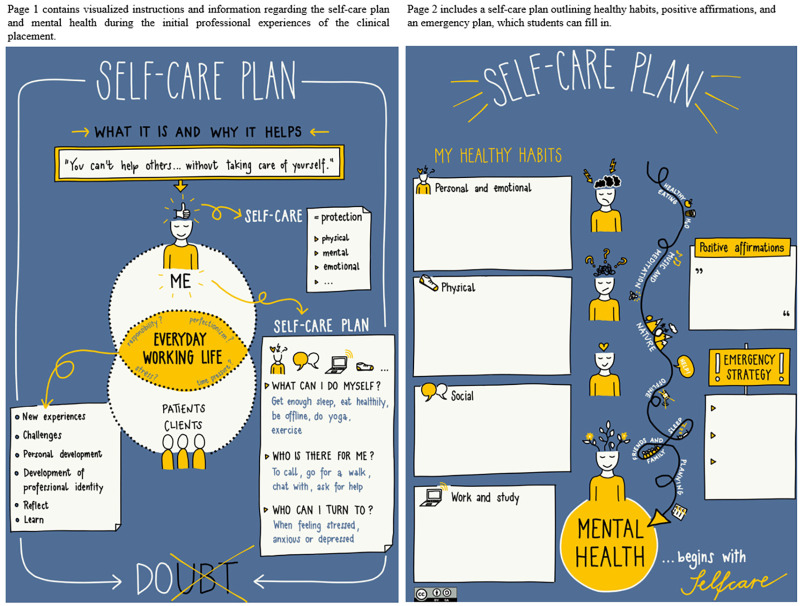
Self-Care Plan Template for Students to Complete.

Development and implementation process:

1. Consultation with international expert:
  The BFH-DPT expert group engaged in detailed discussions with the international expert, who provided valuable insights into the integration of self-care strategies, such as the self-care plan. The international expert consulted for the self-care plan is a professor specializing in working with physiotherapy students engaged in clinical placements in rehabilitation within mental health care. Their expertise encompasses supporting the mental health of healthcare students, particularly physiotherapy students, by providing guidance, mentorship, and evidence-based strategies during their clinical training. This support is tailored to address the unique challenges faced by students in mental health rehabilitation settings, including balancing academic and clinical demands, managing stress, and fostering resilience. The international expert shared examples of self-care plans used in their institution, which served as a foundational reference for the intervention at BFH-DPT.
2. Adaptation and customization:
  Based on the insights gained, the BFH-DPT expert group adapted the self-care plan to align with the specific needs and context of their students. This involved tailoring the content to focus on the challenges faced by physiotherapy students, such as managing academic stress, coping with the demands of clinical placements and perfectionism, and fostering resilience.
3. Components of the self-care plan (Figure 2):
  This self-care plan (OER - open educational ressource) provides students with a structured way to reflect on and improve their mental well-being. Focusing on healthy habits across personal, emotional, physical, social, and academic domains, it helps students identify actionable strategies to support their holistic health. Students should be able to recognize warning signs of stress or burnout and proactively implement coping strategies before issues escalate. Including a crisis plan is particularly helpful, as it allows students to prepare steps to follow in emergency situations, promoting a sense of security and preparedness.
4. Implementation strategy:
  We used two approaches for implementation:

a. Instructional video approach: a video was created to introduce the self-care plan to students, explaining its importance and providing guidance on self-reflection and how to incorporate the plan into their daily routines. The video featured a variety of examples designed to enhance physical, emotional, and social well-being. The main elements included:

- Physical well-being: encouragement of regular exercise, proper nutrition, and adequate sleep.
- Emotional well-being: mindfulness exercises, journaling, and relaxation techniques to manage stress and anxiety.
- Social well-being: strategies to maintain social connections and seek support from peers and mentors.

b. PDF guide: a detailed PDF document was provided to students, serving as a guide on how to develop and maintain their self-care practices. This guide included templates for planning daily activities, setting goals, and reflecting on progress.

5. Integration into the curriculum:
  The self-care plan was integrated into the curriculum at different stages, with Cohort 1 introduced to the plan before their second clinical placement and Cohort 2 before the start of their first clinical placement.

### Survey Development Process

To develop a relevant and tailored survey, the Mental Health Professionals Stress Scale (MHPSS)^33^ was initially used as a foundation and model. However, since the MHPSS did not fully capture the specific issues we aimed to investigate among our students, an expert group decided to develop a survey specifically tailored to our program. This process involved adapting and refining the questionnaire to ensure it was relevant and effectively addressed the unique needs and challenges faced by BFH-DPT students.

Regarding the reliability and validity of the survey, it is important to note that no psychometric testing was conducted. The primary focus was on developing a customized self-care plan tailored to the specific needs of BFH-DPT students as quickly as possible. Given the urgency to address the rising dropout rates and the mental health challenges exacerbated by the Coronavirus disease (COVID-19) pandemic, the expert group prioritized the immediate implementation of the survey and subsequent interventions over conducting extensive psychometric evaluations. Consequently, while the survey was carefully designed to be relevant and useful, its psychometric properties, such as reliability and validity, were not formally tested.

### Survey

The survey included the following areas: self-care, perfectionism, self-doubt, and idealized image of everyday hospital life (Table 1). The survey was developed by the staff of the study support department of the bachelor's program. The survey was completed by an open, voluntary meeting where students were invited to provide personal input and suggestions for the curriculum to optimize and support self-care.

**Table 1.** Overview of the Survey.

A. Questions on Self-Care	Question Type and Likert Scale Answers
A1 Do you know what a self-care plan is?	1-2 (1 = yes, 2 = no)
A2: Do you have a self-care plan?	1-2 (1 = yes, 2 = no)
A3: Evaluate this statement for the following topics: I was able to speak freely with the mentor about Mental stress Physical stress Self-doubt Sorrows/worries	1-4 (1 = completely disagree / 4 = completely agree)
A4: What helped you cope with everyday work?	1-4 (1 = sports; 2 = conversations, 3 = nature/creative activities, 4 = being offline)
A5: It was easy for me to process personal patient stories/fates?	1-4 (1 = completely disagree / 4 = completely agree)
A6: Would you like to create a self-care plan?	1-2 (1 = yes, 2 = no)
B. Questions on perfectionism	
B1: I felt like I had to do everything perfectly.	1-4 (1 = completely agree/ 4 = completely disagree)
B2: I have high expectations of myself in relation to exam results.	1-4 (1 = completely agree/ 4 = completely disagree)
B3: I felt very disappointed having knowledge gaps while working with patients.	1-4 (1 = completely agree/ 4 = completely disagree)
C. Questions on self-doubt	
C1: My professional self-image depends on the objective success of my physiotherapeutic treatment.	1-4 (1 = completely agree / 4 = completely disagree)
C2: I could not deal with self-doubt in my first/third clinical placement?	1-4 (1 = completely agree / 4 = completely disagree)
D. Questions on idealized image of everyday hospital life	
D1: Professional life in practice differed strongly from the ideas you developed in class.	1-4 (1 = completely agree / 4 = completely disagree)
D2: I experienced these differences negatively.	1-4 (1 = completely agree / 4 = completely disagree)
D3: It was difficult for me to make clinical decisions independently.	1-4 (1 = completely agree / 4 = completely disagree)
D4: It was difficult for me to be responsible for the physiotherapy treatment.	1-4 (1 = completely agree / 4 = completely disagree)

A1, D1, and D2 used for Cohort 1 and Cohort 2 after first clinical placement.

### Participants

Participants were recruited through announcements during lectures and emails to students from the undergraduate physiotherapy program from BFH-DPT of two cohorts (n = 192; Cohort 1: n = 91, mean age: 24.6 years; Cohort 2: n = 101, mean age: 22.9 years). Inclusion criteria included being healthy, and a physiotherapy student enrolled in the undergraduate physiotherapy program at BFH-DPT, while exclusion criteria included students from other institutions, programs, or cohorts. Data was collected anonymously.

### Outcome

The primary outcome, defined as self-care, is directly related to the survey conducted. This outcome reflects the key focus of the survey, which assessed self-care alongside related constructs such as perfectionism, self-doubt, and idealized images of everyday hospital life. A survey with three yes/no questions (A1, A2, A6) was used to assess current use of, and interest in, creating and implementing a self-care plan during the first clinical placement (T0) for both cohorts. Two 4-item Likert rating scales were utilized to analyze speaking freely about different stressors and determine the effort coping with mental load, ranging from completely disagree (1) to completely agree (4) (questions A3, A5). Higher scores indicate better self-care and mental health. In addition, participants were asked to indicate their preferred coping strategies, with the option to select multiple responses: sports (1), having conversations (2), being in nature or creative (3), or being offline (4).

Secondary outcomes were perfectionism, self-doubts, and an idealized image of everyday hospital life (Table 2). We used a 4-item Likert rating scale ranging from completely agree (1) to completely disagree (4). Higher scores indicate better management of perfectionism and self-doubt, as well as a more balanced view of the idealized image of everyday hospital life.

**Table 2.** Overview of the Secondary Outcomes.

Target Parameters	Measuring Method	Target
Perfectionism	Scores can range from 1 to 4, lower scores representing higher perfectionism and greater disappointment	Level of perfectionism
Self-doubts	Scores can range from 1 to 4, lower scores represent higher self-doubts.	Perceived self-doubt and professional image
Idealized image of everyday hospital life	Scores can range from 1 to 4, lower scores represent strong, negative differences and more difficulties in everyday hospital life.	Level of difference between idealized expectations and first/third clinical placement experiences
Students’ responsiveness	Retention rate: number of participants completing the survey divided by number of participants enrolled at baseline	> 5%
Data collection & data analysis	Available time of the survey	< 30 days via internet
	Number of measurements errors / inconsistent data	< 5% missing values

### Data Analysis

Descriptive analyses were used to summarize all outcome variables with mean and standard deviation (SD) for continuous variables and percentages (%) for categorical variables. Questions A3 and A4 were analyzed using descriptive statistics only. A binominal test was conducted to assess students’ perceptions of their self-care experience during the third clinical placement. Chi-square analyses were performed to investigate differences between the first and third clinical placement of Cohort 1, as well as differences between Cohort 1 and Cohort 2 after the first clinical placement, with respect to the primary outcome self-care and the secondary outcomes idealized image of everyday hospital life, perfectionism, and self-doubt. An intention to treat analysis was conducted. IBM SPSS Statistics 28.0.1 (IBM Corp. Released 2021. IBM SPSS Statistics for Windows, Version 28.0. Armonk, NY: IBM Corp) was used for all statistical analysis. The level of significance was set at *P* < .05.

## Results

### Students’ Characteristics

A total of 80 out of 192 students completed the survey, resulting in a retention rate of 58.1%. Table 3 presents the characteristics of Cohort 1 and Cohort 2. In Cohort 1, 52 students responded to all questions after the first clinical placement (T0), with a response rate of 57% and 33 students responded after the third clinical placement (T1), with a response rate of 36.3%. In Cohort 2, 47 students completed all questions after the first clinical placement, yielding a response rate of 47.5%.

**Table 3.** Students’ Characteristics for Cohort 1 and Cohort 2.

	Cohort 1	Cohort 2
Mean age (years)	24.6	22.9
Female (n/%)	61 / 68	86 / 86
Male (n/%)	29 / 32	15 / 15
Total responding T0 (n)	52	47
Retention rate (%)	43	53
Total responding T1 (n)	33	
Retention rate (%)	63.8	

T0 represents the measurement (survey) after the first clinical placement, T1 after the third clinical placement.

### Cohort 1 Comparison of First and Third Clinical Placement

#### Outcome Self-Care

**Figure 3.**
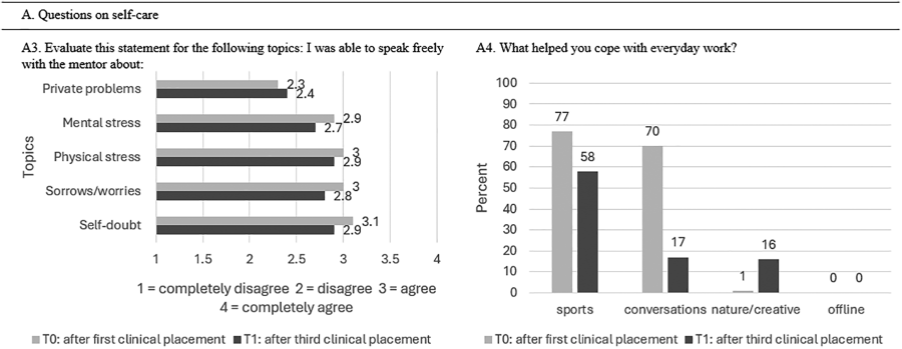
Overview of Students’ Experiences With Self-Care After Their First and Third Clinical Placements.

After the first clinical placement 42% did not know what a self-care plan is and 58% reported knowing what a self-care plan is (question A1). About 38% of students reported using a self-care plan while 62% reported no usage (question A2). Cohort 1 reported during their first and third clinical placement similar agreements in speaking freely with their mentors (question A3; Figure 3). No association with the processing of personal patient stories or fates was determined (question A5; Figure 3). The Pearson chi-square value was 3.91 with degree of freedom (df) of 3, equaling a *P*-value of .271 (Table 4).

**Table 4.** Scores of Questions on Self-Care (n = 52) in Percent (%) and Mean Points (±Standard Deviation (SD)) for the Process of Personal Patient Stories/Fates.

Outcome Self-Care	T0	T1	*P*-Value
A1: Do you know what a self-care plan is?	58% yes		
	42% no		
A2: Do you have a self-care plan?	38% yes	23% yes	.096
	62% no	77% no	
A3: Evaluate this statement for the following topics: I was able to speak freely with the mentor about Mental stress Physical stress Self-doubt Sorrows/worries	See Figure 3.		
A4: What helped you cope with everyday work?	See Figure 3.		
A5: It was easy for me to process personal patient stories/fates?	3.21 (± 0.80)	3.50 (± 0.64)	.271
A6: Would you like to create a self-care plan?	46% yes		
	54% no		

T0 represents the measurement (survey) after the first clinical placement, T1 after the third clinical placement.

A1, A5 measurement only at T0.

During both the first and third clinical placements, sports (T0:77%; T1:58%) and conversations (T0:70%; T1:17%) remained the most important methods for processing the workday. Being offline was not selected as a method by any students during either the first or third clinical placement (0%) (question A4). Students provided personal input on mental health, suggesting that topics related to mental health should be more consistently integrated into the curriculum, including practical applications and strategies to reduce mental load.

#### Outcome Perfectionism

No significant association was found regarding perfectionism. Table 5 represents the mean point score values on the Likert scale after T0 and T1.

**Table 5.** Scores for Perfectionism (n = 52) in Mean Points (±Standard Deviation (SD)).

Qutcome Perfectionism	T0 Mean (± SD)	T1 Mean (± SD)	*P*-Value
B1: I felt like I had to do everything perfectly	1.96 (± 0.69)	1.92 (± 0.48)	.98
B2: I have high expectations of myself in relation to exam results.	1.67 (± 0.51)	1.81 (± 0.56)	.24
B3: I felt very disappointed having knowledge gaps while working with patients.	2.58 (± 0.70)	2.73 (± 0.49)	.14

T0 represents the measurement (survey) after the first clinical placement, T1 after the third clinical placement.

**Figure 4.**
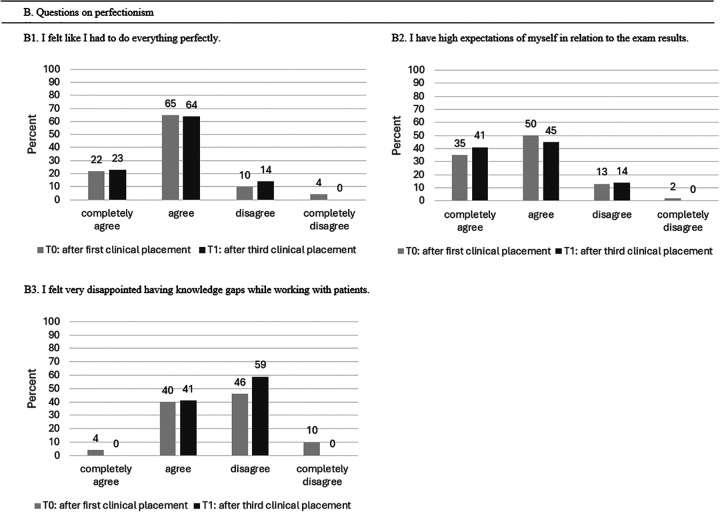
Overview of Students’ Experiences With Perfectionism After Their First and Third Clinical Placements.

Figure 4 represents the overall feeling of perfectionism after the third clinical placement, with 64% of students at T1 (T0: 65%) agreeing that they feel the need to do everything perfectly, and 23% at T1 (T0: 22%) completely agreeing. Similarly, the need to achieve perfection or meet high expectations regarding exam results remained unchanged and equally high (T0: 35% completely agreed; 50% agreed. T1: 41% completely agreed, 45% agreed). During the first clinical placement, 46% of students disagreed with feeling disappointment triggered by gaps in knowledge, while a higher percentage (59%) disagreed during the third clinical placement.

#### Outcome Self-Doubt

**Figure 5.**
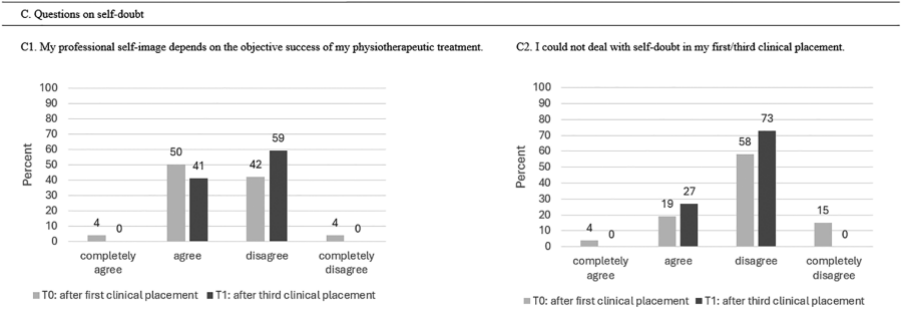
Overview of Students’ Experiences With Self-Doubt After Their First and Third Clinical Placements.

The professional self-image became significantly less dependent on the success of students’ physical therapy treatment from T0 to T1 (Pearson chi-square: 10.9, *P* = .012). Managing self-doubts became significantly easier (C2) in T1 compared with T0 (Pearson chi-square: 14.4, *P* = .001). The mean score values from the Likert scale for self-doubt are presented in Table 6 and Figure 5 represents the overview of students’ experiences with self-doubt after their first and third clinical placements.

**Table 6.** Scores for Self-Doubt (n = 52) in Mean Points (±Standard Deviation (SD)).

Outcome Self-Doubt	T0 Mean (± SD)	T1 Mean (± SD)	*P*-Value
C1. My professional self-image depends on the objective success of the physiotherapeutic treatment.	2.46 (± 0.64)	2.77 (± 0.50)	.012*
C2. I could not deal with self-doubt in my clinical placement.	2.92 (± 0.74)	2.83 (± 0.38)	.001*

T0 represents the measurement (survey) after the first clinical placement, T1 after the third clinical placement.

* *P* < .05.

#### Outcome Idealized Image of Everyday Hospital Life

**Figure 6.**
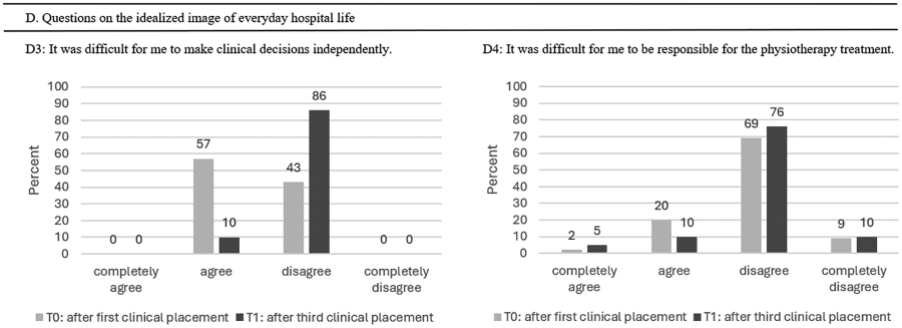
Overview of Students’ Experiences With Idealized Image of Everyday Hospital Life After Their First and Third Clinical Placements.

Table 7 provides an overview of questions D1-D4 how students rate the idealized image of everyday hospital life after their clinical placement using a Likert scale. After the first and third clinical placements, there was significantly less disappointment about the gaps in knowledge when working with patients. Figure 6 illustrates that 86% of the students at T1 disagreed with the statement that clinical decision-making was difficult (Table 7: Pearson chi-square: 93.4, df: 2, *P* < .001). No association was determined between pre and post self-care intervention in student's statement “responsible for the physiotherapy treatment” (Pearson chi-square: 5.4, df: 3, *P* = .145).

**Table 7.** Scores for Idealized Image of Everyday Hospital Life (n = 52) in Mean Points (±Standard Deviation (SD)).

Outcome Idealized Image of Everyday Hospital Life	T0 Mean (± SD)	T1 Mean (± SD)	*P*
D1. Professional life in practice differed strongly from the ideas you developed in class.	2.71 (± 0.75)		
D2. I experienced these differences negatively.	2.75 (± 0.56)		
D3: It was difficult for me to make clinical decisions independently.	2.58 (± 0.50)	3.94 (± 0.24)	.001*
D4: It was difficult for me to be responsible for the physiotherapy treatment.	2.13 (± 0.60)	2.92 (± 0.52)	.145

T0 represents the measurement (survey) after the first clinical placement, T1 after the third clinical placement. Mean points ranged from 1 = completely disagree to 4 = completely agree.

D1 and D2 measurement only at T0.

* *P* < .05.

### Comparison Between the First Survey of Cohort 1 and Cohort 2

#### Outcome Self-Care

A chi-square test was used to compare the self-care outcomes between Cohort 1 and Cohort 2 during the first clinical placement (Table 8). There was a significant association between the cohorts and knowledge about the self-care plan (*P* = .002). Table 9 presents the cross-tabulation.

**Table 8.** Chi-Square Test of Independence for Self-Care and Cohort 1 (n = 52) and Cohort 2 (n = 47).

Question	Chi-Squared	df	*P*-Value
A1. Do you know what a self-care plan is?	9.49	1	.002*
A2. Do you have a self-care plan?	1.10	1	.294
A5. It was easy for me to process personal patient stories/fates?	1.195	3	.754
A6. Would you like to create a self-care plan?	0.22	1	.883

* *P* < .05.

**Table 9.** Cross-Tabulation for the Question “Do You Have a Self-Care Plan?” and Yes/No Answer.

			Group		
Question			Cohort 1	Cohort 2	Total
A2. Do you have a self-care plan?	Yes	Number of students	20	23	43
		% Do you have a self-care plan?	46.5	53.5	100.0
		% group	38.5	48.9	43.4
		% total number	20.2	23.2	43.4
	No	Number	32	24	56
		% Do you have a self-care plan?	57.1	42.9	100.0
		% group	61.5	51.1	56.6
		% total number	32.3	24.2	56.6
Total		Number	52	47	99
		% Do you have a self-care plan?	52.5	47.5	100.0
		% group	100.0	100.0	100.0
		% total number	52.5	47.5	100.0

**Figure 7.**
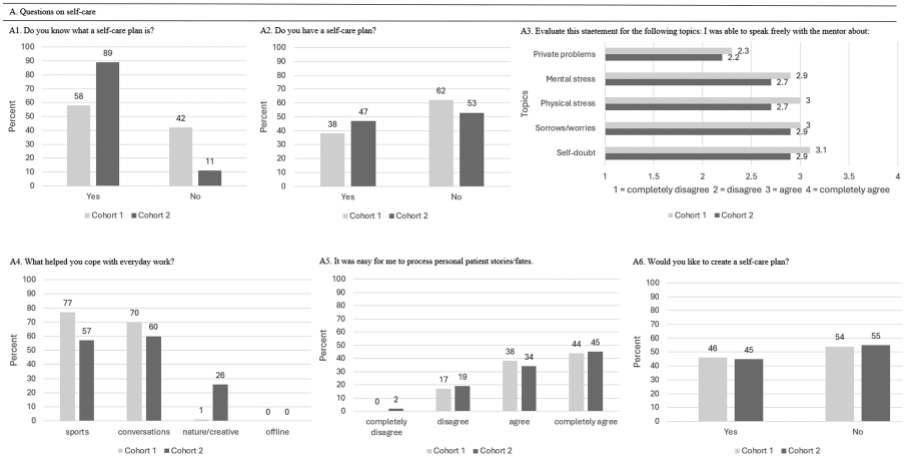
Comparison of Students’ Experiences With Self-Care During the First Clinical Placement Between Cohort 1 and Cohort 2.

Overall, Figure 7 illustrates that coping strategies for managing stressful situations were slightly different in Cohort 1 and 2 (question A4). However, both cohorts predominantly preferred sports (77% of Cohort 1; 57% of Cohort 2) and having conversations with friends and family (70% of Cohort 1; 60% of Cohort 2). Notably, none of the students in both cohorts chose being offline.

#### Outcome Perfectionism

**Figure 8.**
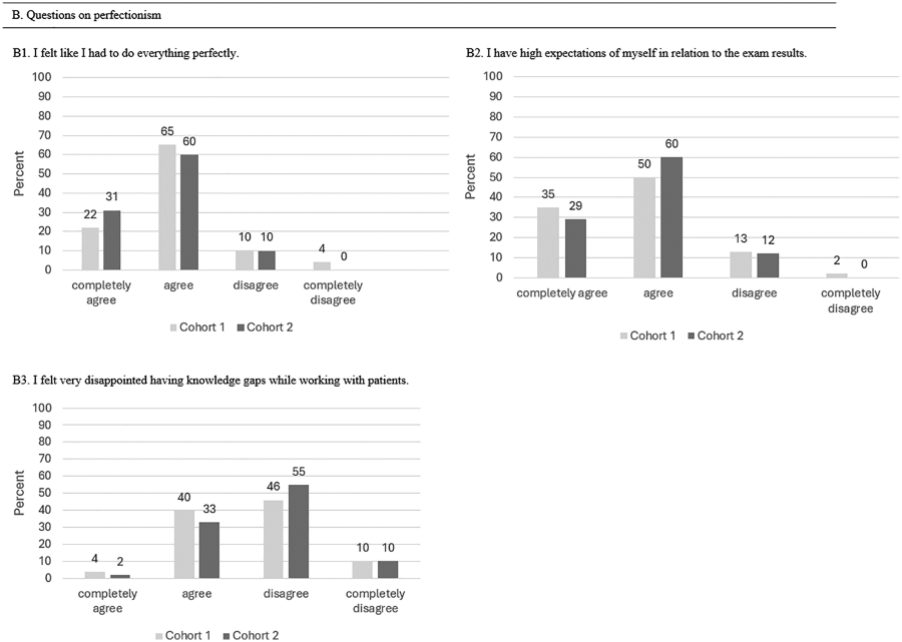
Comparison of Students’ Experiences With Perfectionism During Their First Clinical Placement Between Cohort 1 and Cohort 2.

Figure 8 shows that 22% of the students in Cohort 1 completely agreed, and 65% agreed that they felt the need to do everything perfectly (*P* = .38). These high expectations were also reflected in exam results, with 50% of Cohort 1 and 60% of Cohort 2 agreeing, while 35% of Cohort 1 and 29% of Cohort 2 completely disagreed with having high expectations (question B2). No significant difference was found regarding high expectations related to exam results (*P* = .19). During the first clinical placement, 40% of the students from Cohort 1 and 33% from Cohort 2 reported feeling disappointment triggered by gaps in knowledge (*P* = .77).

#### Outcome Self-Doubt

**Figure 9.**
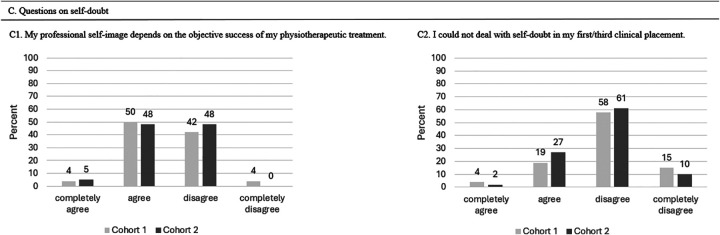
Comparison of Students’ Experiences With Self-Doubt During Their First Clinical Placement Between Cohort 1 and Cohort 2.

Table 10 presents the outcome values of self-doubt between the two cohorts. No significant differences were observed. Figure 9 represents the comparison of students’ experiences with self-doubt during their first clinical placement between Cohort 1 and Cohort 2.

**Table 10.** Mean (± Standard Deviations (SD)) Scores for Self-Doubt (n = 99).

Statement to Be Rated	N (Cohort)	Mean (± SD)	*P*-Value
C1. My professional self-image depends on the objective success of the physiotherapeutic treatment.	52 (1)	2.46 (± 0.64)	.55
	47 (2)	2.45 (± 0.58)	
C2. I could not deal with self-doubt in my first clinical placement.	52 (1)	2.92 (± 0.74)	.38
	47 (2)	2.77 (± 0.63)	

#### Outcome Idealized Image of Everyday Hospital Life

**Figure 10.**
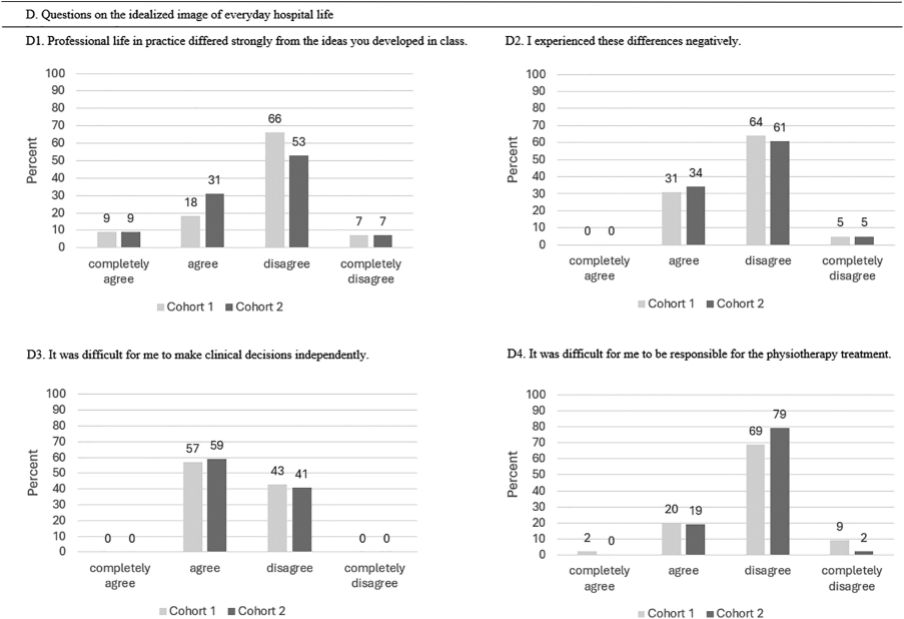
Comparison of Idealized Image of Everyday Hospital Life During Their First Clinical Placement Between Cohort 1 and Cohort 2.

The outcome idealized image of everyday hospital life revealed no differences between Cohort 1 and 2 (Table 11 and Figure 10).

**Table 11.** Mean (± Standard Deviations (SD)) Scores for Idealized Image of Everyday Hospital Life (n = 99).

Statement to Be Rated	N (Cohort)	Mean (± SD)	*P*-Value
D1. Professional life in practice differed strongly from the ideas you developed in class.	52 (1)	2.71 (± 0.75)	.41
	47 (2)	2.57 (± 0.744)	
D2. I experienced these differences negatively.	52 (1)	2.75 (± 0.56)	.902
	47 (2)	2.70 (± 0.55)	
D3. It was difficult for me to make clinical decisions independently.	52 (1)	2.37 (± 0.49)	.691
	47 (2)	2.40 (± 0.50)	
D4. It was difficult for me to be responsible for the physiotherapy treatment.	52 (1)	2.87 (±0.60)	.322
	47 (2)	2.83 (± 0.43)	

## Discussion

This study evaluated the impact of a self-care plan on the mental health of undergraduate BFH-DPT physiotherapy students during their clinical placement. The self-care plan was specifically designed to promote self-efficacy in managing mental health by encouraging students to incorporate daily healthy habits supporting physical, emotional, and social well-being. Prior to the development, an expert with experience in a similar context was consulted. Their insights provided valuable practical perspectives. The resulting adaptation tailored to the specific needs of our student population and presented in a thoughtful, engaging format has remained relevant to this day. A working group was then formed to draw up a development plan. This included the process beginning with the creation of a self-care plan and ending with its implementation in the curriculum. A 6-month timeframe was set and successfully met.

The results present a multifaceted and nuanced landscape. On the one hand, they indicate significant changes or emerging trends; on the other hand, no lasting effects were observed after a one-time use of video instructions designed to promote self-care among undergraduate physiotherapy students at the BFH-DPT. However, it appears that a (single) presentation of a video about the self-care plan is insufficient to foster cognitive learning or self-awareness in undergraduate physiotherapy students at BFH-DPT. Learning is typically defined as relatively permanent change in attitudes, behavior, knowledge, or skills resulting from identifiable psychological or social experiences.^34^ A key characteristic of learning involves permanence: changes are not considered learning if they are temporary.^34^ Although the current study did not show significant effects on students’ mental health, potentially due to the short-term nature of the intervention, a contrasting view is provided by Green.^35^ Her study explored the impact of holistic self-care interventions on nursing students and found notable outcomes, highlighting the potential benefits of such interventions in promoting well-being. Over a 5-week period, the students received education on the importance of self-care in managing the stress associated with their academic and clinical responsibilities. The interventions included both didactic teaching and interactive activities, focusing on various aspects of self-care such as sleep, nutrition, aromatherapy, positive affirmations, and exercise. Upon completion of the program, students reported a decrease in stress levels and an enhanced ability to cope with the stress associated with their nursing studies. Although our study examined similar self-care interventions, variations in duration, intensity, or method may have influenced the outcomes. This aligns with findings that weekly yoga and meditation interventions—forms of self-care—positively affect students’ stress and anxiety levels,^36^ reinforcing the value of integrating holistic self-care strategies within the curriculum.

Alongside these strategies, mentoring is a vital component of the Bachelor of Science in Physiotherapy BFH program, significantly contributing to students’ academic development and professional growth. Results from question A3 indicate that while students are generally able to communicate about mental and physical stress, sorrows, and self-doubt, they appear less willing to address private problems, which could nonetheless represent an important component of self-care. Mentoring has been shown in the literature to positively impact orientation within the field and overall well-being.^37^ Through mentoring relationships, students can identify their strengths and weaknesses, set, and achieve personal goals, and develop effective coping mechanisms to target stress.^38^ Given the demanding nature of healthcare professions,^39,40^ it is essential that both mentors and mentees prioritize self-care to maintain well-being and ensure long-term career success. This commitment to self-care might involve supporting students with self-reflection questions and supporting them with practices such as maintaining a healthy work-life balance, setting well-being goals, and seeking support when needed. According to Ramani et al, peer-to-peer mentoring can be an effective method for exploring various strategies and coping skills to implement self-care management in daily life,^37^ as peers can help identify barriers and discuss ways to overcome them. In summary, reflecting on the students’ needs, the importance of self-care should be more strongly emphasized in the future, as it supports both personal well-being and professional growth in healthcare.

Research shows that well-being and mental health are closely associated with feelings of perfectionism, particularly in professional settings. Martin et al found that self-critical perfectionism uniquely predicted emotional exhaustion and burnout in physician.^41^ Similarly, Collin et al^42^ revealed that maladaptive perfectionism is prevalent among dental students and is associated with increased stress, burnout, and psychological distress. These findings underscore the importance of recognizing and addressing perfectionism to prevent adverse outcomes such as burnout and psychological distress among individuals in various professional domains. Our survey results align with these findings, as perfectionism and self-esteem issues are critical concerns for our undergraduate physiotherapy students. In Cohort 1, 22% of students completely agreed, and 65% agreed that they felt the need to do everything perfectly (*P* = .38). These high expectations were also reflected in exam results, with 50% of Cohort 1 and 60% of Cohort 2 students reporting agreement with having high expectations. Correspondingly, at the end of their first clinical placement (T0), 40% of students in Cohort 1 and 33% in Cohort 2 reported feeling disappointed when they couldn’t optimally design therapy with their current skills. These results indicate that perfectionism seems to be a common issue in undergraduate physiotherapy students and needs to be addressed through awareness-raising and strategies, as it may significantly influence their mental health. As a result, greater support should be offered, especially in the early stages of the project, with mentorship playing a crucial role.

Regarding the idealized image of everyday hospital life, this study identified a significant shift: 86% of students found clinical decision-making easier after the interval between their first and third clinical placements in comparison to attending purely lessons in the classroom (question D3). Additionally, it is intriguing to note the positive development in dealing with self-doubt, as managing self-doubt became significantly easier (question C2) at T1 compared to T0. A key factor in this process may be the role of practicing professionals who guide students’ professional identity through constructive feedback and supervisory support. These changes may be explained by the “transition shock” theory, which was initially applied to nursing students.^43^ The theory describes an individual process encompassing emotional, intellectual, physical, sociocultural, and developmental challenges.^43^ Its structuring includes elements of transition theory, reality shock, culture and acculturation shock as well as theories of professional role adjustment, growth and development and change theory.^43^ Transition shock manifests as the shift from the familiar role of a student to the relatively unfamiliar role of a practicing nurse.^43^ Duchscher et al^43^ described the fears of newly graduated nurses, such as being exposed as clinically incompetent, or struggling to manage assigned roles and responsibilities during this transitional stage. These characteristics can be compared to some of the findings in this study's physiotherapy students, as both cohorts faced similar challenges in making clinical decisions during their first clinical placement. The learning curve and increased experience gained over time may also play a role. However, the idealized image of everyday hospital life did not differ significantly between Cohort 1 and Cohort 2 in questions D1-D4. Golz et al highlighted the importance of raising awareness about the gap between expectations and experiences when entering the professional world, as well as the need to implement self-care and stress management skills in the curriculum.^24^ In line with this, the systematic review by Chiodelli et al^44^ demonstrated the potential of mindfulness-based interventions as effective strategies for promoting mental well-being among undergraduate students in educational settings. Notably, Plummer et al^45^ emphasized that the duration of mindfulness programs, as part of self-care strategies, is crucial for achieving the expected stress-reducing effects and meaningful outcomes. They expressed concern that the once-a-week exposure in their study, where the intervention group participated in a 20-week, 1.5-h-per-week Mindfulness-Based Intervention curriculum, may not have been sufficient in strength or length to significantly impact participants’ stress levels and quality of life.

Therefore, it is evident that there is a need to integrate self-care skills into health profession curricula.^24,45^ In summary, our results highlight the need to reconsider the implementation process of self-care management and how to continue empowering undergraduate physiotherapy students to prioritize their mental health regularly within the curriculum. This focus should not be limited to times of struggle but should be integrated as a regular practice, even during good times.

### Future Perspectives

Increasing contact hours to discuss and practice these strategies may be beneficial to further emphasizing the importance of self-care and mental health among undergraduate physiotherapy students. Moving from theory to practice, this could involve implementing lectures and group sessions where students learn self-care techniques that promote active learning. It is important that students engage deliberately with their own self-care plan. Additionally, training for mentors could be valuable in educating them on how to support and improve mental health among their mentees.

### Limitations

A major limitation is the validity of the survey. The questions were created by staff from the study support department of the BSc program to address specific aspects of the situation for undergraduate physiotherapy students, but they were not validated before using with students. Future studies should use a validated and reliable survey to achieve more robust findings. Another limitation was the low level of student adherence within the study. To enhance adherence in future studies, we recommend providing information on how self-care can positively impact students’ academic and personal lives, as this may increase their motivation to participate.

Another limitation concerns the generalizability of the findings, which is restricted by the single-institution setting and the relatively small sample size. Since all participants were undergraduate physiotherapy students from the same program, the results may not be representative of students in other institutions, academic disciplines, or cultural contexts.

The retention rate of 58.1% reflects several potential factors that may have influenced participation. One possibility is the timing of the survey, which coincided with busy academic or clinical schedules, potentially leading to lower engagement. Previous research has shown that classroom experiences directly impact student success and retention.^46^ Next Australian universities,^47^ as well as Rogan et al^48^ and Schenk et al^49^ from Bern University of Applied Sciences have highlighted that the workload for undergraduate students in physiotherapy education is very high, which contributes to academic stress. Another consideration could be survey fatigue, as students may have been asked to complete multiple surveys throughout their training, reducing motivation to participate. Additionally, some students may not have perceived the survey's relevance to their immediate needs or concerns. These factors could explain why a significant proportion of students declined to participate. This understanding of contributing factors should be considered when planning future studies, which could aim to improve participation by addressing these barriers, for example, by scheduling surveys during less intensive periods or emphasizing their relevance to students’ education and well-being.

## Conclusion

This study explored the impact of a self-care plan on the mental health of BFH-DPT physiotherapy students during clinical placement. Although the intervention was thoughtfully designed and contextually adapted, results indicate that a single exposure to video-based instruction is insufficient to produce lasting changes in self-awareness or mental well-being. A more sustained and integrated approach to self-care education across the curriculum is necessary. Extending self-care training could better equip students to manage stress and adapt to the demands of clinical placements. Embedding self-care as a core component of physiotherapy education may strengthen students’ coping skills and promote long-term mental health and professional success.

Nonetheless, the self-care plan marks an important step in promoting healthy habits. It may encourage students to reflect on and plan practices such as balanced nutrition, regular physical activity, sufficient rest, social connection, and mindfulness. These strategies aim to enhance students’ resilience and well-being throughout their education.

## Supplemental Material

sj-docx-1-mde-10.1177_23821205251374552 - Supplemental material for Development and Implementation of a Self-Care Plan for an Undergraduate Physiotherapy Curriculum in Switzerland: A Survey Study Supplemental material, sj-docx-1-mde-10.1177_23821205251374552 for Development and Implementation of a Self-Care Plan for an Undergraduate Physiotherapy Curriculum in Switzerland: A Survey Study by Simone Zingg, Jorina Janssens, Irene Koenig, Patricia Wassmer, Angela Blasimann and Slavko Rogan in Journal of Medical Education and Curricular Development
