# Supplementary material for: Development and Implementation of a Self-Care Plan for an Undergraduate Physiotherapy Curriculum in Switzerland: A Survey Study
Source: J Med Educ Curric Dev. 2025 Sep 15;12:23821205251374552. doi: 10.1177/23821205251374552 (PMC12437181; doi:10.1177/23821205251374552)
Supplement: sj-docx-1-mde-10.1177_23821205251374552 - Supplemental material for Development and Implementation of a Self-Care Plan for an Undergraduate Physiotherapy Curriculum in Switzerland: A Survey Study [file sj-docx-1-mde-10.1177_23821205251374552.docx]

**Revised Standards for Quality Improvement Reporting Excellence (SQUIRE 2.0) September 15, 2015**

| **Text Section and Item Name** | **Section or Item Description** | **We have added an additional column here in reference to the article by Zingg et al. in order to insert our statement.**  **ZINGG ET AL** |
| --- | --- | --- |
| **Notes to authors** | - The SQUIRE guidelines provide a framework for reporting new knowledge about how to improve healthcare - The SQUIRE guidelines are intended for reports that describe [system](#_bookmark13) level work to improve the quality, safety, and value of healthcare, and used methods to establish that observed outcomes were due to the [intervention(s)](#_bookmark8). - A range of approaches exists for improving healthcare. SQUIRE may be adapted for reporting any of these. - Authors should consider every SQUIRE item, but it may be inappropriate or unnecessary to include every SQUIRE element in a particular manuscript. - The SQUIRE Glossary contains definitions of many of the key words in SQUIRE. - The Explanation and Elaboration document provides specific examples of well-written SQUIRE items, and an in-depth explanation of each item. - Please cite SQUIRE when it is used to write a manuscript. |  |
| **Title and Abstract** |  |  |
| **1. Title** | Indicate that the manuscript concerns an [initiative](#_bookmark6) to improve healthcare (broadly defined to include the quality, safety, effectiveness, patient- centeredness, timeliness, cost, efficiency, and equity of healthcare) | “Development and implementation of a self-care plan for an undergraduate physiotherapy curriculum in Switzerland: a survey study“ |
| **2. Abstract** | 1. Provide adequate information to aid in searching and indexing 2. Summarize all key information from various sections of the text using the abstract format of the intended publication or a structured summary such as: background, local [problem,](#_bookmark10) methods, interventions, results, conclusions | Please see page 2 |
| **Introduction** | *Why did you start?* |  |
| [**3. Problem**](#_bookmark10) [**Description**](#_bookmark10) | Nature and significance of the local [problem](#_bookmark10) | Please refer to lines 130–141. |
| **4. Available knowledge** | Summary of what is currently known about the [problem,](#_bookmark10) including relevant previous studies | Please see lines 78–128 for the identified stressors prevalent among physiotherapy students and their coping strategies. |

| **5.** [**Rationale**](#_bookmark12) | Informal or formal frameworks, models, concepts, and/or [theories](#_bookmark14) used to explain the [problem,](#_bookmark10) any reasons or [assumptions](#_bookmark0) that were used to develop the [intervention(s),](#_bookmark8) and reasons why the [intervention(s)](#_bookmark8) was expected to work | Please refer to line 210 for details on the development and implementation process of the self-care plan intervention within the physiotherapy bachelor’s program. |
| --- | --- | --- |
| **6. Specific aims** | Purpose of the project and of this report | The aims are as follows, please see line 143: “Consequently, the purpose of this study was to investigate the impact of the self-care plan on the mental health of undergraduate BFH-DPT physiotherapy students during their clinical placement.“ |
| **Methods** | *What did you do?* |  |
| **7.** [**Context**](#_bookmark1) | Contextual elements considered important at the outset of introducing the [intervention(s)](#_bookmark8) | Contextual elements related to stressors and existing gaps in self-care within current health professions education institutions are described. Please refer to line 111 for details. |
| **8.** [**Intervention(s)**](#_bookmark8) | 1. Description of the [intervention(s)](#_bookmark8) in sufficient detail that others could reproduce it 2. Specifics of the team involved in the work | The development and implementation process of the intervention is clearly described. Furthermore, the structure of the curriculum and the role of mentors in addressing self-care are well outlined in the sections *Methods – Physiotherapy and the Mentoring Program in the BSc Physiotherapy Curriculum* and *Study Design*. |
| **9. Study of the Intervention(s)** | 1. Approach chosen for assessing the impact of the [intervention(s)](#_bookmark8) 2. Approach used to establish whether the observed outcomes were due to the [intervention(s)](#_bookmark8) | Please refer to line 189: “The objective of the study was to administer a survey to Cohort 1 following their first clinical placement (prior to receiving any instruction on self-care planning) and again after their third placement (subsequent to receiving such instruction). The responses from these two time points within Cohort 1 were then analyzed and compared. In contrast, Cohort 2 completed the survey only after their first clinical placement, having received self-care plan instruction in advance. This design enabled a comparative analysis between the post-placement responses of Cohort 2 — with prior exposure to self-care planning — and those of Cohort 1 following their initial placement without such preparation.” |
| **10. Measures** | 1. Measures chosen for studying [processes](#_bookmark11) and outcomes of the [intervention(s)](#_bookmark8), including rationale for choosing them, their operational definitions, and their validity and reliability 2. Description of the approach to the ongoing assessment of contextual elements that contributed to the success, failure, efficiency, and cost 3. Methods employed for assessing completeness and accuracy of data | 1. The survey development process is described starting at line 257, the outcomes from line 288, and the data analysis from line 306. 2. / c) The problem regarding the retention rate is addressed in the discussion starting at line 627. |
| **11. Analysis** | 1. Qualitative and quantitative methods used to draw [inferences](#_bookmark5) from the data 2. Methods for understanding variation within the data, including the effects of time as a variable | Data analysis is included from line 306 onward as descriptive analyses were conducted for all outcomes, with a binomial test evaluating self-care perceptions and Pearson chi-square tests comparing cohorts and clinical placement timing. |
| **12. Ethical**  **Considerations** | [Ethical aspects](#_bookmark2) of implementing and studying the [intervention(s)](#_bookmark8) and how they were addressed, including, but not limited to, formal ethics review and potential conflict(s) of interest | Please see Statements and Declarations => Ethical considerations |
| **Results** | *What did you find?* |  |
| **13. Results** | 1. Initial steps of the [intervention(s)](#_bookmark8) and their evolution over time (*e.g.*, time-line diagram, flow chart, or table), including modifications made to the intervention during the project 2. Details of the [process](#_bookmark11) measures and outcome 3. Contextual elements that interacted with the [intervention(s)](#_bookmark8) 4. Observed associations between outcomes, interventions, and relevant contextual elements 5. Unintended consequences such as unexpected benefits, problems, failures, or costs associated with the [intervention(s).](#_bookmark8) 6. Details about missing data | 1. An overview is provided in Figure 1. 2. The Development and implementation process of the self-care plan is stated on page 8.  - process measures: retention rate is stated - Outcome measures: The outcome measures were defined in the survey. The primary outcome was self-care. To assess this, a survey with three yes/no questions (items A1, A2, A6) was used to measure current engagement with and interest in developing and applying a self-care plan during the first clinical placement (T0) for both cohorts. - Secondary outcomes included perfectionism, self-doubt, and an idealized image of everyday hospital life. These were assessed using a 4-item Likert scale ranging from 1 = strongly agree to 4 = strongly disagree.  1. A statement about the retention rate is provided on page 26. “The retention rate of 58.1% reflects several potential factors that may have influenced participation.” 2. The results for “Cohort 1 comparison: first and third clinical placement” as well as “Comparison between the first survey of Cohort 1 and Cohort 2” are presented using descriptive analyses. These were used to summarize all outcome variables, reporting means and standard deviations (SD) for continuous variables, and percentages (%) for categorical variables. Items A3 and A4 were analyzed using descriptive statistics only. |
| **Discussion** | *What does it mean?* |  |
| **14. Summary** | 1. Key findings, including relevance to the [rationale](#_bookmark12) and specific aims 2. Particular strengths of the project | 1. Key findings related to the specific aim, as well as the development and implementation process, are summarized in lines 501-510. 2. See lines 643–645 for reference. |

| **15. Interpretation** | 1. Nature of the association between the [intervention(s)](#_bookmark8) and the outcomes 2. Comparison of results with findings from other publications 3. Impact of the project on people and [systems](#_bookmark13) 4. Reasons for any differences between observed and anticipated outcomes, including the influence of [context](#_bookmark1) 5. Costs and strategic trade-offs, including [opportunity costs](#_bookmark9) | 1. The video-based instruction and structured implementation of a self-care plan were associated with improved student knowledge and awareness of self-care practices. The observed changes in survey responses between Cohort 1 and Cohort 2 suggest a positive relationship, although causal conclusions are limited by the study design (please see line 416/417) 2. This point has been addressed in the discussion section. 3. We have integrated into the conclusion that the project had a positive impact on students by fostering early awareness of personal well-being, and that future steps should focus on further implementation of the self-care plan and its broader integration into the curriculum. 4. This was discussed, for example, with regard to the retention rate and its underlying reasons starting at line 627. 5. - |
| --- | --- | --- |
| **16. Limitations** | 1. Limits to the [generalizability](#_bookmark3) of the work 2. Factors that might have limited [internal validity](#_bookmark7) such as confounding, bias, or imprecision in the design, methods, measurement, or analysis 3. Efforts made to minimize and adjust for limitations | 1. This point has been addressed in the limitations section. 2. The use of a non-validated survey may have reduced measurement precision and internal validity; this issue has been addressed in the limitations section 3. To mitigate limitations, the same data collection instruments and procedures were applied consistently across both cohorts. This design enabled a comparative analysis between the post-placement responses of Cohort 2—who had prior exposure to self-care planning—and those of Cohort 1 following their initial placement without such preparation. Limitations were transparently acknowledged in the discussion, and findings were interpreted with appropriate caution. |
| **17. Conclusions** | 1. Usefulness of the work 2. Sustainability 3. Potential for spread to other [contexts](#_bookmark1) 4. Implications for practice and for further study in the field 5. Suggested next steps | 1. The project demonstrated clear usefulness by enhancing students’ awareness and knowledge of self-care strategies during clinical placements. It provided a structured approach that supported students in managing stress and reflecting on their well-being, addressing an often-neglected aspect of healthcare education. 2. The intervention’s sustainability is supported by its integration into the existing curriculum and the use of reusable video-based materials, which require minimal ongoing resources 3. Given its flexible design and focus on universally relevant topics such as self-care and stress management, the program has strong potential for adaptation and adoption in other healthcare education settings and disciplines. 4. And e) These points are addressed in the Future Perspectives section |
| **Other information** |  |  |
| **18. Funding** | Sources of funding that supported this work. Role, if any, of the funding organization in the design, implementation, interpretation, and reporting | Funding statement:  No external funding was obtained for this study (page 28). |

**Table 2. Glossary of key terms used in SQUIRE 2.0. This Glossary provides the intended meaning of selected words and phrases as they are used in the SQUIRE 2.0 Guidelines. They may, and often do, have different meanings in other disciplines, situations, and settings.**

**Assumptions**

Reasons for choosing the activities and tools used to bring about changes in healthcare services at the [system](#_bookmark13) level.

# Context

Physical and sociocultural makeup of the local environment (for example, external environmental factors, organizational dynamics, collaboration, resources, leadership, and the like), and the interpretation of these factors (“sense-making”) by the healthcare delivery professionals, patients, and caregivers that can affect the effectiveness and [generalizability](#_bookmark3) of [intervention(s)](#_bookmark8).

# Ethical aspects

The value of [system](#_bookmark13)-level [initiatives](#_bookmark6) relative to their potential for harm, burden, and cost to the stakeholders. Potential harms particularly associated with efforts to improve the quality, safety, and value of healthcare services include [opportunity costs](#_bookmark9), invasion of privacy, and staff distress resulting from disclosure of poor performance.

# Generalizability

The likelihood that the [intervention(s)](#_bookmark8) in a particular report would produce similar results in other settings, situations, or environments (also referred to as external validity).

# Healthcare improvement

Any systematic effort intended to raise the quality, safety, and value of healthcare services, usually done at the [system](#_bookmark13) level. We encourage the use of this phrase rather than “quality improvement,” which often refers to more narlinely defined approaches.

# Inferences

The meaning of findings or data, as interpreted by the stakeholders in healthcare services – improvers, healthcare delivery professionals, and/or patients and families

# Initiative

A broad term that can refer to organization-wide programs, narlinely focused projects, or the details of specific interventions (for example, planning, execution, and assessment)

# Internal validity

Demonstrable, credible evidence for efficacy (meaningful impact or change) resulting from introduction of a specific intervention into a particular healthcare [system.](#_bookmark13)

# Intervention(s)

The specific activities and tools introduced into a healthcare [system](#_bookmark13) with the aim of changing its performance for the better. Complete description of an intervention includes its inputs, internal activities, and outputs (in the form of a logic model, for example), and the mechanism(s) by which these components are expected to produce changes in a [system’s](#_bookmark13) performance.

# Opportunity costs

Loss of the ability to perform other tasks or meet other responsibilities resulting from the diversion of resources needed to introduce, test, or sustain a particular [improvement](#_bookmark4) initiative

# Problem

Meaningful disruption, failure, inadequacy, distress, confusion or other dysfunction in a healthcare service delivery [system](#_bookmark13) that adversely affects patients, staff, or the [system](#_bookmark13) as a whole, or that prevents care from reaching its full potential

# Process

The routines and other activities through which healthcare services are delivered

# Rationale

Explanation of why particular [intervention(s)](#_bookmark8) were chosen and why it was expected to work, be sustainable, and be replicable elsewhere.

# Systems

The interrelated structures, people, [processes](#_bookmark11), and activities that together create healthcare services for and with individual patients and populations. For example, systems exist from the personal self- care system of a patient, to the individual provider-patient dyad system, to the microsystem, to the macrosystem, and all the way to the market/social/insurance system. These levels are nested within each other.

# Theory or theories

Any “reason-giving” account that asserts causal relationships between variables (causal theory) or that makes sense of an otherwise obscure [process](#_bookmark11) or situation (explanatory theory). Theories come in many forms, and serve different purposes in the phases of [improvement](#_bookmark4) work. It is important to be explicit and well-founded about any informal and formal theory (or theories) that are used.
